# Migration status, country of origin and long-term outcomes in multiple sclerosis: a Swedish nationwide study

**DOI:** 10.1136/jnnp-2026-338466

**Published:** 2026-03-23

**Authors:** Anna Karin Hedström, Tomas Olsson, Lars Alfredsson

**Affiliations:** 1 Department of Clinical Neuroscience, Karolinska Institutet, Stockholm, Sweden; 2 Institute of Environmental Medicine, Karolinska Institutet, Stockholm, Sweden; 3 Centre for Occupational and Environmental Medicine, Region Stockholm, Stockholm, Sweden

**Keywords:** MULTIPLE SCLEROSIS, Prognosis

## Abstract

**Background:**

Multiple sclerosis (MS) shows marked geographic and ethnic variation, but the contribution of migration status and region of origin to long-term outcomes remains uncertain. We aimed to examine disability, cognitive and patient-reported outcomes across these dimensions.

**Methods:**

We analysed 4008 individuals with incident relapsing-onset MS from two nationwide Swedish case-control studies (2005–2019), with registry-based follow-up through April 2022. Repeated measures of expanded disability status scale (EDSS), symbol digit modalities test (SDMT) and MS impact scale (MSIS-29) were obtained prospectively. Cox regression compared outcomes by migration status (Swedish-born with Swedish parents, first-generation immigrants, Swedish-born with immigrant parent/s) and region of origin (Sweden, Nordic countries, non-Nordic Europe, Middle East and North Africa (MENA) and other).

**Results:**

Compared with Swedish-born individuals with Swedish parents, first-generation immigrants had higher risks of confirmed disease worsening (CDW) (HR 1.36, 95% CI 1.18 to 1.56) and reaching EDSS 3 (HR 1.25, 95% CI 1.03 to 1.52), whereas Swedish-born individuals with immigrant parents did not differ from the reference group. By region of origin, participants of MENA origin had higher risks of CDW (HR 1.45, 95% CI 1.15 to 1.82), EDSS 3 (HR 1.46, 95% CI 1.07 to 2.00), EDSS 4 (HR 1.82, 95% CI 1.19 to 2.80), SDMT worsening (HR 1.79, 95% CI 1.26 to 2.51) and MSIS-29 worsening.

**Conclusions:**

Within a universal-access health system, first-generation immigration into Sweden was associated with faster disability progression, and MENA origin showed consistent excess risks across disability, cognitive and patient-reported outcomes. The overall pattern points to a combination of migration-related and origin-linked influences on MS progression, supporting early risk stratification and culturally adapted care strategies.

**WHAT IS ALREADY KNOWN ON THIS TOPIC**

- Previous studies suggest that multiple sclerosis (MS) incidence and outcomes vary by ethnicity and geography, but findings on disease progression by migration status and region of origin are inconsistent and often confounded by socioeconomic factors and access to care. Few studies have disentangled immigration from parental origin or examined disability, cognitive and patient-reported outcomes simultaneously in a universal-access healthcare setting.

**WHAT THIS STUDY ADDS**

- In a nationwide Swedish cohort with comparable access to specialist care and disease-modifying therapies, first-generation immigrants, but not Swedish-born individuals with immigrant parents, had faster disability progression than Swedish-born individuals with Swedish parents. Individuals of Middle East and North Africa (MENA) origin showed consistently higher risks of disability progression, cognitive worsening and patient-related impact, indicating both migration-related and origin-linked influences on MS progression.

**HOW THIS STUDY MIGHT AFFECT RESEARCH, PRACTICE OR POLICY**

- These findings support early risk stratification and sustained monitoring of immigrants and patients of MENA origin, even within equitable healthcare systems. They also highlight the need for research into biological, environmental and sociocultural mechanisms underlying progression disparities and for culturally adapted strategies to improve long-term MS outcomes.

## Background

 Multiple sclerosis (MS) is a chronic inflammatory and neurodegenerative disease with substantial heterogeneity in disease activity and long-term progression. Global patterns show clear latitudinal gradients in incidence and prevalence, alongside racial/ethnic disparities often attributed to UV exposure, vitamin D status, infections and lifestyle.^1^,^3^ Although numerous studies have assessed outcomes across racial/ethnic groups,^4^,^9^ comparatively few have disentangled migration status from parental origin or systematically examined disability progression, cognitive and patient-reported outcomes across these strata. Some cohorts suggest more aggressive disease in specific origin groups,^4^,^6^ whereas others find little or no independent association after accounting for socioeconomic factors and access to care.^8 9^ Inconsistencies are likely to reflect differences in case mix, outcome definitions, follow-up intensity and the extent to which migration status was separated from parental origin. It is unclear to what extent observed differences, when present, reflect genetic background versus modifiable exposures or healthcare-system factors.

Sweden provides a valuable setting to address these questions. Over recent decades, it has experienced substantial immigration from Nordic and other European countries as well as from the Middle East and North Africa (MENA), within a tax-funded system offering near-universal access to specialist neurology and modern disease-modifying therapies.^10 11^ This combination enables evaluation of progression disparities while minimising confounding by financial barriers to care.

We investigated whether the risk of disability progression differs by migration status and region of origin in a nationwide Swedish MS cohort and secondarily assessed cognitive and patient-reported worsening. Our aim was to provide policy-relevant estimates that inform both mechanistic hypotheses and equitable care.

## Methods

Our study population was drawn from two population-based case-control studies in Sweden.^12 13^ The epidemiological investigation of multiple sclerosis (EIMS) recruited incident MS cases from hospital neurology units between April 2005 and December 2019 (n=3567). The response rate among cases was 93% for EIMS. We excluded individuals without EDSS follow-up in the Swedish MS Registry (n=202), those with progressive-onset MS (n=180) and those with unknown disease phenotype.

The genes and environment in the MS study identified 6148 cases from the Swedish MS Registry between November 2009 and November 2011 (case response rate 82%). To ensure the cohort reflected recently diagnosed rather than long-standing disease, we restricted inclusion to relapsing-remitting MS patients recruited within 5 years of diagnosis (n=823). All cases were diagnosed by neurologists according to the McDonald criteria.^14^ After applying these criteria, the analytic sample comprised 4008 participants (online supplemental eFigure 1).

A diagnosis of MS in Sweden was not an inclusion criterion. A small number of immigrants (n=5) had received their diagnosis prior to migration, but were included if they had prospective follow-up in the Swedish MS Registry.

## Data collection

Baseline demographic, clinical and lifestyle information was obtained at study inclusion via standardised questionnaires. Our primary exposures were migration status and region of origin. Migration status was classified as Swedish-born with Swedish parents, first-generation immigrants and Swedish-born with immigrant parent(s). For first-generation immigrants, we derived the time between immigration and baseline. Region of origin was based on the participants’ and parents’ countries of birth and grouped as Sweden, Nordic countries, Europe (excluding Nordic), MENA and other. In individuals with mixed origins, a hierarchical classification was applied to reduce exposure heterogeneity. Participants were categorised as Swedish if the participant and both parents were Swedish-born, as Nordic if at least one was Nordic-born and none were born outside the Nordic countries, as European (excluding the Nordic countries) if at least one was born in Europe and none were born outside Europe, and as MENA if the participant or at least one parent was born in MENA. The remaining combinations were classified as Other. The MENA background was prioritised in the classification, given its potential to reflect a distinct genetic background and migration-related exposures compared with Nordic and other European origins.

The following variables were included as covariates: educational level (pre-secondary education, secondary education or post-secondary education), smoking (current smokers vs non-smokers), snuff use (yes or no), past infectious mononucleosis (yes, no or unsure), body mass index (BMI <30 vs >30 kg/m^2^), sun exposure habits, regular fish consumption, alcohol use and physical activity. Sun exposure combined three items on ultraviolet radiation exposure, each scored 1–4, into an index from 3 (lowest) to 12 (highest). Oily fish consumption was recorded on a 4-point scale and dichotomised into regular (1–3 times/month or more) or non-regular. Alcohol use was reported as weekly intake of beer, wine, fortified wine and liquor and converted to grams/week. Physical activity was reported in four leisure-time levels and dichotomised into regular (exercise >1–2 times/week with sweating) or non-regular (predominantly sedentary activities or moderate activities without sweating). For patients recruited between 2005 and 2009, serum 25-hydroxy-vitamin D (ng/mL) was measured with a chemiluminescent immunoassay (Diasorin AB, Sweden) equimolarly detecting 25-hydroxy-vitamin D_2_ and D_3_.

## Outcome measures

Clinical data were retrieved by linkage to the Swedish MS registry,^11^ a nationwide quality-of-care register with prospectively recorded assessments, including repeated expanded disability status scale (EDSS) scores, symbol digit modalities test (SDMT) scores and MS impact scale (MSIS-29) scores.

Baseline was defined as the date of the first recorded EDSS. The primary outcome was confirmed disability worsening (CDW), defined as an increase in the EDSS score by at least one point from baseline, sustained between two follow-up visits separated in time by no <6 months (or by 1.5 points if EDSS at baseline was 0, and 0.5 points if the baseline EDSS ≥5.5). Analyses of time to reach EDSS 3 and EDSS 4, confirmed at a second visit ≥6 months later, were restricted to participants with a baseline EDSS <3. Complementary outcomes were cognitive worsening, defined as a decrease of 8 points or more on the SDMT,^15^ and patient-reported worsening on the MSIS-29, defined as an increase of 8.5 units or more on the 0–100 transformed physical and psychological subscales.^16^ EDSS is routinely recorded at clinical visits and recorded in the Swedish MS Register. SDMT and MSIS-29 are collected during the same visits but were not available for all patients, resulting in smaller analytic samples for these outcomes.

## Statistical analysis

We used Cox proportional hazards models to examine the association of migration status and region of origin with the prespecified outcomes, and we report HRs with 95% CIs. Time at risk was calculated from baseline until the event, loss to follow-up, death or end of follow-up (6 April 2022), whichever occurred first. The proportional hazard assumption was tested using Schoenfeld residuals and showed no violations.

Analyses were adjusted for age at baseline, sex, duration between disease onset and baseline, calendar year of diagnosis, baseline EDSS and DMT exposure, captured as both initial DMT category (none, low-efficacy and high-efficacy) and the proportion of follow-up time on DMT. In a sensitivity analysis, we further adjusted for visit frequency, educational level, past IM, smoking status, snuff use, alcohol consumption, obesity, regular fish intake, sun exposure habits and physical activity. Among first-generation immigrants, we evaluated residence time in Sweden as a categorical exposure (<5, 5–10 or >10 years from immigration to baseline), using Swedish-born individuals with Swedish parents as the reference. To evaluate whether associations with migration status and region of origin were independent of each other, we constructed models that included both variables simultaneously by creating eight mutually exclusive categories defined by combinations of migration status and region of origin, using Swedish-born with Swedish parents as the reference. All analyses were conducted in SAS V.9.4.

## Results

The cohort comprised 4008 individuals: Swedish-born with Swedish parents (n=3160), first-generation immigrants (n=401) and Swedish-born with immigrant parent/s (n=447). Both immigrant groups had a younger age at onset than Swedish-born with Swedish parents (p<0.0001). Indicators of care access and treatment exposure were largely similar across groups. Baseline EDSS was comparable (p=0.11), while baseline SDMT was lower among first-generation immigrants (p<0.0001). First-generation immigrants and Swedish-born with immigrant parents had slightly more frequent visits per year compared with Swedish-born with Swedish parents, although absolute differences were small. Overall, we found no evidence of reduced access to care among immigrants, whereas lifestyle and baseline cognition differed (table 1).

**Table 1:** Baseline demographics, clinical and lifestyle characteristics by migration status at baseline

Characteristic	Swedish-born with Swedish parents	First-generation immigrants	Swedish-born with immigrant parent/s	P value
N	3160	401	447	
Nordic countries Europe MENA Other	- - - -	84 (20.4) 146 (35.4) 106 (25.7) 65 (18.5)	171 (38.3) 159 (35.6) 51 (11.4) 66 (14.8)	
Age at disease onset (SD)	34.4 (10.4)	32.5 (10.5)	31.1 (9.1)	<0.0001
Female, n (%)	2277 (72.1)	281 (70.1)	310 (69.4)	0.39
University studies, n (%)	1215 (38.5)	153 (38.2)	184 (41.2)	0.53
Time to diagnosis (SD)	2.9 (4.1)	3.0 (3.9)	2.5 (3.5)	0.04
Mean disease duration, years (SD)	3.7 (6.0)	3.6 (5.8)	2.8 (4.4)	0.009
Mean follow-up time, years (SD)	10.8 (4.7)	10.4 (4.6)	10.1 (4.4)	0.001
Never treated, n (%) LET, n (%) LET and HET, n (%) HET, n (%)	275 (8.7) 1091 (34.5) 1219 (38.6) 575 (18.2)	24 (6.0) 136 (33.9) 173 (43.1) 68 (17.0)	34 (7.6) 140 (31.3) 182 (40.7) 91 (20.4)	0.15
Time to first DMT (SD)	0.5 (1.7)	0.4 (1.3)	0.3 (1.2)	0.18
Prop of follow-up on DMT (SD)	0.9 (0.2)	0.9 (0.2)	0.9 (0.2)	0.20
Baseline EDSS (SD)	1.8 (1.5)	1.8 (1.4)	1.7 (1.3)	0.11
Baseline SDMT (SD)	52.3 (11.9)	48.8 (13.9)	52.6 (11.7)	<0.0001
Yearly visits (SD)	1.3 (0.6)	1.3 (0.7)	1.4 (0.6)	0.007
Yearly EDSS (SD)	1.0 (0.5)	1.1 (0.6)	1.1 (0.5)	0.001
Past IM, n (%) No past IM, n (%) Unsure, n (%)	595 (18.8) 2177 (68.9) 388 (12.3)	46 (11.5) 274 (68.3) 81 (20.2)	76 (17.0) 300 (67.1) 71 (15.9)	0.30
Current smokers, n (%)	714 (22.6)	112 (27.9)	130 (29.1)	0.001
Snuff use, n (%)	452 (14.3)	38 (9.5)	62 (13.9)	0.03
Alcohol consumption, n (%)	2081 (65.9)	208 (51.9)	263 (58.8)	<0.0001
Mean BMI, kg/m^2^ (SD)	25.5 (10.3)	25.6 (12.7)	25.0 (5.4)	0.22
Obesity; BMI >30, n (%)	862 (27.3)	103 (25.7)	121 (27.1)	0.58
Regular fish intake, n (%)	2403 (76.0)	284 (70.8)	317 (70.9)	0.009
Sun exposure	6.1 (1.8)	6.2 (1.8)	6.3 (1.8)	0.05
Vit D level, ng/mL (SD)	63.2 (27.1)	51.1 (24.6)	62.4 (25.4)	<0.0001
Physical activity score (SD)	2.4 (0.9)	2.2 (1.0)	2.4 (1.0)	0.0006
Regular exercise, n (%)	1244 (39.4)	132 (32.9)	182 (40.7)	0.03

Follow-up is defined as the time from baseline to the last recorded visit or the administrative end of the follow-up period. Vitamin D was measured in a subsample (n=1083).

BMI, body mass index; DMT, disease-modifying therapy; EDSS, Expanded Disability Status Scale; HET, high-efficacy therapy; IM, infectious mononucleosis; LET, low-efficacy therapy; MENA, Middle East and North Africa; SDMT, Symbol Digits Modalities Test.

First-generation Immigrants had a higher risk of CDW (HR 1.36, 95% CI 1.18 to 1.56) and EDSS 3 (HR 1.25, 95% CI 1.03 to 1.52) compared with Swedish-born individuals with Swedish parents. Swedish-born with immigrant parent/s did not differ from the reference group for any endpoint (table 2).

**Table 2:** Disability progression by migration status

Confirmed disability worsening					
	N	Years (SD)	Outcome (%)	HR (95% CI)*	HR (95% CI)†
Swedish-born with Swedish parents	3149	6.4 (4.5)	1548 (49.2)	1.0 (reference)	1.0 (reference)
First-generation immigrants	412	5.6 (4.3)	231 (56.1)	**1.32 (1.15 to 1.52)**	**1.36 (1.18 to 1.56)**
Swedish-born with immigrant parent/s	447	6.2 (4.3)	212 (47.4)	1.06 (0.92 to 1.23)	1.08 (0.93 to 1.45)
EDSS 3					
	N	Years (SD)	Outcome (%)	HR (95% CI)*	HR (95% CI)†
Swedish-born with Swedish parents	2438	7.5 (4.8)	781 (32.0)	1.0 (reference)	1.0 (reference)
First-generation immigrants	317	6.7 (4.7)	115 (36.3)	**1.29 (1.06 to 1.57)**	**1.25 (1.03 to 1.52)**
Swedish-born with immigrant parent/s	376	7.3 (4.7)	118 (31.4)	1.08 (0.89 to 1.32)	1.07 (0.88 to 1.30)
EDSS 4					
	N	Years (SD)	Outcome (%)	HR (95% CI)*	HR (95% CI)†
Swedish-born with Swedish parents	2438	8.8 (4.7)	781 (27.0)	1.0 (reference)	1.0 (reference)
First-generation immigrants	317	8.4 (4.7)	115 (30.3)	1.19 (0.88 to 1.59)	1.13 (0.84 to 1.52)
Swedish-born with immigrant parent/s	376	8.7 (4.7)	118 (28.4)	1.12 (0.84 to 1.48)	1.08 (0.81 to 1.43)

Significant hazard ratios (HRs) are shown in bold (p<0.05)

* Adjusted for age and sex.

† Adjusted for age, sex, disease duration, baseline EDSS and disease-modifying therapy exposure.

.EDSS, Expanded Disability Status Scale.

Age at onset differed markedly by origin (p<0.0001) and was lowest in MENA. Sex distribution also varied (p=0.03). Baseline EDSS and indicators of care access and treatment exposure were similar across groups. Treatment regimen and proportion of follow-up on DMT differed statistically (p=0.02 and p=0.007) but with small absolute differences. Baseline SDMT was lower in MENA (p<0.0001), and several lifestyle factors varied (table 3).

**Table 3:** Baseline demographics, clinical and lifestyle characteristics by regional origin

	Sweden	Nordic countries	Europe	MENA	Other	P value
N	3160	255	305	157	131	
Age at disease onset (SD)	34.4 (10.4)	34.6 (10.0)	32.4 (9.3)	27.4 (8.7)	29.6 (8.7)	<0.0001
Female, n (%)	2277 (72.1)	192 (75.3)	205 (67.2)	99 (63.1)	95 (72.5)	0.03
University studies, n (%)	1215 (38.5)	104 (40.8)	130 (42.6)	52 (33.1)	51 (38.9)	0.33
Time to diagnosis (SD)	2.9 (4.0)	2.8 (3.7)	2.8 (3.7)	2.7 (3.8)	2.3 (3.3)	0.14
Mean disease duration, years (SD)	3.7 (6.0)	3.5 (5.4)	3.6 (5.8)	3.0 (4.3)	2.4 (3.7)	0.08
Mean follow-up time, years (SD)	10.8 (4.7)	10.4 (4.5)	10.3 (4.4)	9.6 (4.6)	10.6 (4.3)	0.002
Never treated, n (%) LET, n (%) LET and HET, n (%) HET, n (%)	275 (8.7) 1091 (34.5) 1219 (38.6) 575 (18.2)	21 (8.2) 100 (39.2) 93 (36.5) 41 (16.1)	18 (5.9) 94 (30.8) 133 (43.6) 60 (19.7)	7 (4.5) 48 (30.6) 73 (46.5) 29 (18.5)	12 (8.7) 34 (26.0) 56 (42.8) 29 (22.1)	0.02
Time to first DMT (SD)	0.5 (1.7)	0.4 (1.2)	0.3 (1.3)	0.4 (1.4)	0.4 (1.1)	0.59
Prop of follow-up on DMT (SD)	0.9 (0.3)	0.8 (0.3)	0.9 (0.2)	0.9 (0.2)	0.9 (0.2)	0.007
Baseline EDSS (SD)	1.8 (1.5)	1.8 (1.4)	1.8 (1.4)	1.8 (1.4)	1.6 (1.3)	0.72
Baseline SDMT (SD)	52.2 (11.9)	51.6 (12.3)	51.4 (13.3)	47.4 (13.3)	52.6 (12.2)	0.0001
Yearly visits (SD)	1.3 (0.6)	1.3 (0.6)	1.3 (0.6)	1.4 (0.6)	1.4 (0.8)	0.009
Yearly EDSS (SD)	1.0 (0.5)	1.0 (0.5)	1.1 (0.5)	1.2 (0.6)	1.2 (0.6)	0.0001
Past IM, n (%) No past IM, n (%) Unsure, n (%)	595 (18.8) 2177 (68.9) 388 (12.3)	48 (18.8) 166 (65.1) 41 (16.1)	41 (13.4) 214 (70.2) 50 (16.4)	16 (10.2) 105 (66.9) 36 (22.9)	17 (13.0) 89 (67.9) 25 (19.1)	0.41
Current smokers, n (%)	714 (22.6)	73 (28.6)	82 (26.9)	51 (32.5)	36 (27.5)	0.005
Snuff use, n (%)	452 (14.3)	32 (12.6)	38 (12.5)	15 (9.6)	15 (11.5)	0.35
Alcohol consumption, n (%)	2081 (65.9)	155 (60.8)	183 (60.0)	61 (38.9)	72 (55.0)	<0.0001
Mean BMI, kg/m^2^ (SD)	25.1 (5.0)	25.5 (5.1)	24.7 (5.2)	24.9 (6.0)	25.0 (5.8)	0.92
Obesity; BMI >30, n (%)	862 (27.3)	80 (31.4)	71 (23.3)	40 (25.5)	33 (25.2)	0.28
Regular fish intake, n (%)	2403 (76.0)	190 (74.5)	214 (70.2)	96 (61.2)	101 (77.1)	0.0002
Sun exposure	6.1 (1.8)	6.0 (1.8)	6.5 (1.9)	6.4 (1.7)	6.1 (1.7)	0.007
Vit D level, ng/mL (SD)	63.2 (27.0)	63.4 (28.4)	55.5 (22.7)	44.8 (24.6)	54.2 (24.6)	<0.0001
Physical activity score (SD)	2.4 (0.9)	2.4 (0.9)	2.3 (1.0)	2.2 (1.1)	2.3 (1.1)	0.07
Regular exercise, n (%)	1244 (39.4)	100 (39.2)	112 (36.7)	51 (32.5)	51 (38.9)	0.46

Follow-up is defined as the time from baseline to the last recorded visit or the administrative end of the follow-up period. Vitamin D was measured in a subsample (n=1131).

BMI, body mass index; DMT, disease-modifying therapy; EDSS, Expanded Disability Status Scale; HET, high-efficacy therapy; IM, infectious mononucleosis; LET, low-efficacy therapy; SDMT, Symbol Digits Modalities Test.

Compared with Sweden, MENA had a higher risk of CDW (HR 1.45, 95% CI 1.15 to 1.82), EDSS 3 (HR 1.46, 95% CI 1.07 to 2.00) and EDSS 4 (HR 1.82, 95% CI 1.19 to 2.80). Europe had a modest elevation of CDW only (HR 1.21, 95% CI 1.03 to 1.42), while Nordic and Other were not significantly different from Sweden across endpoints (table 4). MENA also had higher risks of SDMT worsening (HR 1.79, 95% CI 1.26 to 2.51), physical worsening (HR 1.66, 95% CI 1.29 to 2.12) and psychological worsening (HR 1.44, 95% CI 1.14 to 1.82). The Other group showed increased risk of SDMT worsening (HR 2.02, 95% CI 1.46 to 2.79) and psychological worsening (HR 1.36, 95% CI 1.06 to 1.75), while Nordic countries and Europe did not significantly differ from Sweden (table 5).

**Table 4:** Disability progression by regional origin

Confirmed disability worsening					
Origin	N	Years (SD)	Outcome (%)	HR (95% CI)*	HR (95% CI)†
Sweden	3160	6.4 (4.5)	1555 (49.2)	1.0 (reference)	1.0 (reference)
Nordic countries	255	6.0 (4.2)	132 (51.8)	1.11 (0.93 to 1.32)	1.11 (0.93 to 1.33)
Europe	305	5.9 (4.4)	161 (52.8)	**1.19 (1.01 to 1.39)**	**1.21 (1.03 to 1.42)**
MENA	157	5.5 (4.4)	78 (49.7)	**1.33 (1.06 to 1.68)**	**1.45 (1.15 to 1.82)**
Other	131	6.1 (4.4)	65 (49.6)	1.18 (0.92 to 1.51)	1.20 (0.93 to 1.54)
EDSS 3					
Origin	N	Years (SD)	Outcome (%)	HR (95% CI)*	HR (95% CI)†
Sweden	2449	7.5 (4.8)	785 (32.1)	1.0 (reference)	1.0 (reference)
Nordic countries	211	7.1 (4.8)	76 (36.0)	1.14 (0.90 to 1.45)	1.08 (0.85 to 1.36)
Europe	240	7.3 (4.7)	74 (30.8)	1.04 (0.82 to 1.33)	1.06 (0.83 to 1.34)
MENA	124	6.1 (4.6)	42 (33.9)	**1.52 (1.11 to 2.08)**	**1.46 (1.07 to 2.00)**
Other	107	7.3 (4.7)	37 (34.6)	1.28 (0.92 to 1.78)	1.23 (0.89 to 1.72)
EDSS 4					
Origin	N	Years (SD)	Outcome (%)	HR (95% CI)*	HR (95% CI)†
Sweden	2449	8.8 (4.7)	365 (14.9)	1.0 (reference)	1.0 (reference)
Nordic countries	211	8.6 (4.8)	36 (17.1)	1.14 (0.81 to 1.60)	1.07 (0.76 to 1.51)
Europe	240	9.0 (4.6)	30 (12.5)	0.89 (0.61 to 1.29)	0.87 (0.60 to 1.27)
MENA	124	7.4 (4.4)	23 (18.6)	**1.93 (1.26 to 2.96)**	**1.82 (1.19 to 2.80)**
Other	107	9.0 (4.8)	16 (15.0)	1.23 (0.74 to 2.03)	1.16 (0.70 to 1.92)

Significant hazard ratios (HRs) are shown in bold (p<0.05).

* Adjusted for age and sex.

† Adjusted for age, sex, disease duration, baseline EDSS and disease-modifying therapy exposure.

.EDSS, Expanded Disability Status Scale; MENA, Middle East and North Africa.

**Table 5:** Cognitive and patient-reported worsening by regional origin

Cognitive worsening (SDMT decrease by ≥8 points)					
Origin	N	Time (SD)	Outcome (%)	HR (95% CI)*	HR (95% CI)†‡
Sweden	2493	5.5 (3.3)	571 (22.9)	1.0 (reference)	1.0 (reference)
Nordic countries	192	5.7 (3.5)	46 (24.0)	1.03 (0.76 to 1.39)	1.03 (0.76 to 1.39)
Europe	262	5.7 (3.6)	72 (27.5)	1.27 (0.99 to 1.62)	1.27 (0.99 to 1.62)
MENA	145	5.4 (3.4)	36 (24.8)	**1.85 (1.31 to 2.61)**	**1.79 (1.26 to 2.51)**
Other	106	5.6 (3.6)	40 (37.7)	**2.00 (1.45 to 2.77)**	**2.02 (1.46 to 2.79)**
Physical worsening (MSIS-29 physical component increase by ≥8.5 units)					
Origin	N	Time (SD)	Outcome (%)	HR (95% CI)*	HR (95% CI)†§
Sweden	2431	5.1 (4.5)	1014 (41.7)	1.0 (reference)	1.0 (reference)
Nordic countries	189	5.5 (5.7)	74 (39.2)	0.91 (0.72 to 1.15)	0.93 (0.74 to 1.18)
Europe	257	5.0 (4.5)	117 (45.5)	1.14 (0.94 to 1.38)	1.17 (0.96 to 1.41)
MENA	134	4.1 (3.4)	70 (52.2)	**1.70 (1.33 to 2.17)**	**1.66 (1.29 to 2.12)**
Other	104	5.0 (3.6)	50 (48.1)	1.22 (0.92 to 1.63)	1.26 (0.95 to 1.68)
Psychological worsening (MSIS-29 psychological component increase by ≥8.5 units)					
Origin	N	Time (SD)	Outcome (%)	HR (95% CI)*	HR (95% CI)†¶
Sweden	2431	4.8 (4.7)	1204 (49.5)	1.0 (reference)	1.0 (reference)
Nordic countries	189	5.1 (5.6)	86 (45.5)	0.88 (0.70 to 1.09)	0.95 (0.76 to 1.18)
Europe	257	4.8 (4.3)	136 (53.3)	1.04 (0.87 to 1.25)	1.11 (0.93 to 1.33)
MENA	134	3.9 (3.0)	80 (59.7)	**1.39 (1.11 to 1.75)**	**1.44 (1.14 to 1.82)**
Other	104	4.6 (3.8)	64 (61.5)	1.24 (0.99 to 1.60)	**1.36 (1.06 to 1.75)**

Significant hazard ratios (HRs) are shown in bold (p<0.05).

* Adjusted for age and sex.

† Adjusted for age, sex, disease duration, baseline EDSS and disease-modifying therapy exposure.

‡ Adjusted for baseline SDMT.

§ Adjusted for baseline MSIS-29 physical component.

¶ Adjusted for baseline MSIS-29 psychological component.

MENA, Middle East and North Africa; MSIS, Multiple Sclerosis Impact scale; SDMT, Symbol Digit Modalities Test.

Further adjustment for visit frequency, education and lifestyle variables (smoking, snuff use, alcohol use, obesity, regular physical activity, sun exposure and regular fish consumption) attenuated some estimates, but did not alter the overall pattern of associations (online supplemental eTables 1 and 2). Among first-generation immigrants, CDW risk decreased with longer residence in Sweden, yet remained significantly elevated even beyond 10 years (online supplemental eTable 3). In the model combining immigration status and region of origin, patterns were consistent with the main analyses, although some estimates were imprecise due to small numbers (online supplemental eTable 4).

## Discussion

In this nationwide cohort of incident relapsing-onset MS, we observed clear disparities in disease progression by migration status and region of origin, despite comparable access to specialist care and DMT exposure. Compared with Swedish-born individuals with Swedish parents, first-generation immigrants had a higher risk of disability progression, whereas Swedish-born individuals with immigrant parents did not differ from the reference group. When stratified by region of origin, the most consistent excess risks were observed among individuals of MENA origin, who also displayed worse cognitive and patient-reported outcomes.

Previous studies of ethnicity or origin and MS progression have reported mixed results,^4^,^9^ likely reflecting heterogeneity in case mix, outcome definitions, follow-up intensity and whether migration status was separated from parental origin. Our results help reconcile these inconsistencies by showing that disparities persist even when indicators of care access and treatment exposure are comparable across groups. Similar time to diagnosis, onset-to-DMT time intervals, DMT exposure and assessment frequency indicate that unequal access to evaluation or treatment is unlikely to account for the observed disparities.

Among first-generation immigrants, the excess risk was greatest soon after arrival and attenuated with longer residence, yet remained evident more than 10 years later. Exposures that accompany relocation and resettlement, along with post-migration stressors, can plausibly affect inflammation, health behaviours, treatment adherence^17^,^20^ and may partly resolve as individuals adapt. The elevated risks among Swedish-born individuals of MENA origin indicate that origin-linked determinants, such as genetic architecture, vitamin D biology or sociocultural context, contribute beyond migration history.^21^,^23^

Although the region of origin is an imperfect proxy for genetic background, differences in polygenic risk, human leukocyte antigen (HLA) architecture and pharmacogenomic profiles could plausibly influence inflammatory activity, neurodegeneration or treatment response.^22^,^24^ In parallel, exposures correlated with origin, such as infection history, diet and sun exposure, may shape immune regulation and neuroplasticity from early life into adulthood.^25^,^27^ In our cohort, MENA participants had lower vitamin D and distinct lifestyle profiles. Adjustment for lifestyle-related factors attenuated some estimates without eliminating the associations, consistent with partial mediation or residual confounding. Beyond origin-specific exposures, migration itself may involve additional environmental and psychosocial stressors related to resettlement and adaptation, which could influence disease progression. Intergenerational differences may also reflect variation in life-course exposure timing, as Swedish-born individuals with immigrant parents are more likely to have been exposed to the Swedish environment from early life, potentially contributing to the similar progression rates observed in this group. Baseline SDMT was lower in immigrants, particularly those of MENA origin, indicating cognitive vulnerability at diagnosis. Given that subsequent cognitive worsening and MSIS-physical worsening were also increased, measurement artefact alone is unlikely to account for these findings.

Although several studies have reported increased MS incidence among immigrants from MENA regions^28 29^ and, in Canada, increasing risk with longer residence,^30^ these findings concern disease susceptibility rather than progression. Our results, therefore, address a distinct phase of MS and should not be interpreted as contradictory to incidence patterns.

This study has several strengths, including incident cases, registry linkage, repeated clinical, cognitive, patient-reported assessments and analysis within a universal-access setting that minimises financial barriers. Nonetheless, some limitations warrant caution. Region of origin categories combine heterogeneous populations, which may dilute or mask subgroup differences. Some strata were small, yielding imprecise estimates with wide CIs. Despite extensive adjustment, residual confounding from social or environmental factors cannot be excluded.

Clinically, these results argue for early risk stratification and proactive management in newly arrived patients. Since disparities persist beyond 10 years, sustained attention is required. For patients of MENA origin in particular, closer follow-up, systematic cognitive monitoring and a lower threshold for treatment escalation may be justified, while remaining individualised. More generally, equal access to care does not automatically translate into equal outcomes. Interventions that address health behaviours, treatment adherence and culturally mediated barriers to care may be necessary.

In summary, within a universal-access health system, immigration into Sweden was associated with faster disability progression, especially early after arrival, and MENA origin showed consistent excess risks across disability, cognitive and physical patient-reported outcomes. The overall pattern points to a combination of migration-related and origin-linked influences on MS progression and supports targeted strategies for early identification and management of higher-risk patients.

## Supplementary material

10.1136/jnnp-2026-338466 online supplemental file 1

## Data Availability

Data are available upon reasonable request.
