# Supplementary material for: Migration status, country of origin and long-term outcomes in multiple sclerosis: a Swedish nationwide study
Source: J Neurol Neurosurg Psychiatry. 2026 Mar 23;97(7):e338466. doi: 10.1136/jnnp-2026-338466 (PMC13288959; doi:10.1136/jnnp-2026-338466)

eTable 1. Disability progression by migration status.

Confirmed disability worsening				
	N	Years (SD)	Outcome (%)	HR (95% CI)
Swedish-born with Swedish parents	3149	6.4 (4.5)	1548 (49.2)	1.0 (reference)
First-generation immigrants	412	5.6 (4.3)	231 (56.1)	**1.33 (1.16-1.53)**
Swedish-born with immigrant parent/s	447	6.2 (4.3)	212 (47.4)	1.06 (0.92-1.23)
EDSS 3				
	N	Years (SD)	Outcome (%)	HR (95% CI)
Swedish-born with Swedish parents	2438	7.5 (4.8)	781 (32.0)	1.0 (reference)
First-generation immigrants	317	6.7 (4.7)	115 (36.3)	**1.22 (1.00-1.49)**
Swedish-born with immigrant parent/s	376	7.3 (4.7)	118 (31.4)	1.06 (0.87-1.30)
EDSS 4				
	N	Years (SD)	Outcome (%)	HR (95% CI)
Swedish-born with Swedish parents	2438	8.8 (4.7)	781 (27.0)	1.0 (reference)
First-generation immigrants	317	8.4 (4.7)	115 (30.3)	1.05 (0.78-1.42)
Swedish-born with immigrant parent/s	376	8.7 (4.7)	118 (28.4)	1.04 (0.78-1.39)

EDSS=Expanded Disability Status Scale; SD=standard deviation; HR=hazard ratio. Adjustment for age, sex, disease duration, baseline EDSS, DMT exposure, smoking, snuff use, alcohol use, obesity, regular fish intake, sun exposure habits, and regular physical activity.

eTable 2. Confirmed disability progression by region of origin.

Region of origin	N	Time (SD)	CDW (%)	HR (95% CI)
Sweden	3160	6.4 (4.5)	1555 (49.2)	1.0 (reference)
Nordic countries	255	6.0 (4.2)	132 (51.8)	1.11 (0.94-1.34)
Europe	305	5.9 (4.4)	161 (52.8)	**1.18 (1.00-1.40)**
MENA	157	5.5 (4.4)	78 (49.7)	**1.39 (1.10-1.76)**
Other	131	6.1 (4.4)	65 (49.6)	1.17 (0.91-1.51)

EDSS=Expanded Disability Status Scale; SD=standard deviation. Hazard ratios (HRs) adjusted for age, sex, disease duration, baseline EDSS, DMT exposure, smoking, snuff use, alcohol use, obesity, regular fish intake, sun exposure habits, and regular physical activity.

eTable 3. Confirmed disability progression by residence in Sweden.

Time since immigration	N	Years (SD)	CDW (%)	HR (95% CI)
Swedish-born	3607	6.4 (4.5)	1767 (49.0)	1.0 (reference)
<5 years	65	5.0 (4.2)	39 (60.0)	**2.09 (1.36-3.23)**
5-10 years	59	5.8 (4.5)	33 (55.9)	**1.67 (1.11-2.50)**
>10 years	277	5.6 (4.4)	152 (54.9)	**1.27 (1.09-1.48)**

CDW=confirmed disability progression; SD=standard deviation. Hazard ratios (HRs) adjusted for age, sex, disease duration, baseline EDSS, DMT exposure

eTable 4. Disability progression by combined migration status and region of origin.

	Confirmed disability worsening				
Swedish-born	Parents origin	N	Years (SD)	Outcome (%)	HR (95% CI)
+	Sweden	3160	6.4 (4.5)	1555 (49.2)	1.0 (reference)
+	Nordic countries	171	6.0 (4.3)	85 (49.7)	1.08 (0.87-1.35)
+	Europe	159	6.3 (4.4)	74 (46.5)	1.02 (0.80-1.28)
+	MENA	51	5.3 (3.7)	24 (47.1)	**1.52 (1.01-2.28)**
+	Other	66	6.9 (4.4)	29 (43.9)	1.01 (0.70-1.45)
-	Nordic countries	84	5.9 (4.2)	47 (56.0)	1.17 (0.87-1.56)
-	Europe	146	5.5 (4.2)	87 (59.6)	**1.44 (1.07-1.86)**
-	MENA	106	5.5 (4.7)	54 (50.9)	**1.41 (1.07-1.85)**
-	Other	65	5.3 (4.3)	36 (55.4)	**1.43 (1.02-1.98)**
	EDSS 3				
Swedish-born	Parents origin	N	Years (SD)	Outcome (%)	HR (95% CI)
+	Sweden	2449	7.5 (4.8)	785 (32.1)	1.0 (reference)
+	Nordic countries	144	7.4 (4.9)	46 (31.9)	1.00 (0.74-1.35)
+	Europe	134	7.6 (4.7)	39 (29.1)	0.95 (0.70-1.31)
+	MENA	41	6.0 (4.1)	13 (31.7)	**1.74 (1.00-3.03)**
+	Other	57	7.5 (4.5)	20 (35.1)	1.25 (0.80-1.95)
-	Nordic countries	67	6.5 (4.4)	30 (44.8)	1.21 (0.84-1.75)
-	Europe	106	7.1 (4.8)	35 (33.0)	1.20 (0.85-1.69)
-	MENA	83	6.1 (4.8)	29 (34.9)	1.36 (0.94-1.97)
-	Other	50	7.1 (5.0)	17 (34.0)	1.21 (0.75-1.96)
	EDSS 4				
Swedish-born	Parents origin	N	Years (SD)	Outcome (%)	HR (95% CI)
+	Sweden	2449	8.8 (4.7)	365 (14.9)	1.0 (reference)
+	Nordic countries	144	8.7 (5.0)	20 (13.9)	0.93 (0.60-1.46)
+	Europe	134	9.1 (4.6)	17 (12.7)	0.85 (0.52-1.38)
+	MENA	41	7.7 (3.9)	7 (17.1)	**2.11 (1.00-4.53)**
+	Other	57	8.6 (4.6)	12 (21.1)	**1.81 (1.01-3.23)**
-	Nordic countries	67	8.4 (4.3)	16 (23.9)	1.31 (0.79-2.16)
-	Europe	106	8.7 (4.8)	13 (12.3)	0.91 (0.52-1.58)
-	MENA	83	7.3 (4.6)	16 (19.3)	**1.73 (1.04-2.86)**
-	Other	50	9.4 (5.0)	4 (8.0)	0.76 (0.59-1.30)

Reference: Swedish-born with Swedish parents. EDSS=Expanded Disability Status Scale; SD=standard deviation. Hazard ratios (HRs) adjusted for age, sex, disease duration, baseline EDSS, and DMT exposure.

eFigure 1. Flow of participants through the study.

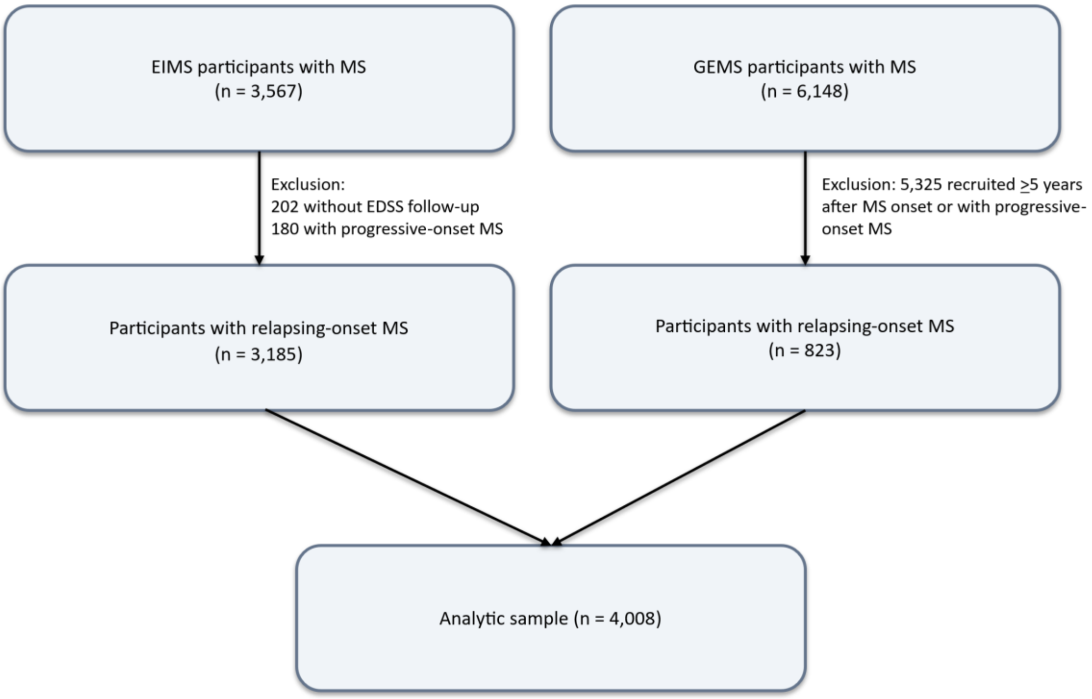

Supplement: online supplemental file 1 [file jnnp-97-7-s001.pdf]
